# GDF15 is an epithelial-derived biomarker of idiopathic pulmonary fibrosis

**DOI:** 10.1152/ajplung.00062.2019

**Published:** 2019-08-21

**Authors:** Yingze Zhang, Mao Jiang, Mehdi Nouraie, Mark G. Roth, Tracy Tabib, Spencer Winters, Xiaoping Chen, John Sembrat, Yanxia Chu, Nayra Cardenes, Rubin M. Tuder, Erica L. Herzog, Changwan Ryu, Mauricio Rojas, Robert Lafyatis, Kevin F. Gibson, John F. McDyer, Daniel J. Kass, Jonathan K. Alder

**Affiliations:** ^1^Dorothy P. and Richard P. Simmons Center for Interstitial Lung Disease, University of Pittsburgh, Pittsburgh, Pennsylvania; ^2^Division of Pulmonary, Allergy, and Critical Care Medicine, University of Pittsburgh, Pittsburgh, Pennsylvania; ^3^Division of Rheumatology and Clinical Immunology, University of Pittsburgh, Pittsburgh, Pennsylvania; ^4^Department of Human Genetics, University of Pittsburgh, Pittsburgh, Pennsylvania; ^5^Division of Pulmonary Sciences and Critical Care Medicine, University of Colorado, Denver, Colorado; ^6^Yale ILD Center of Excellence, Yale University, New Haven, Connecticut; ^7^The Third Xiangya Hospital, Central South University, Changsha, China

**Keywords:** aging, MIC-1, NAG-1, SASP

## Abstract

Idiopathic pulmonary fibrosis (IPF) is the most common and devastating of the interstitial lung diseases. Epithelial dysfunction is thought to play a prominent role in disease pathology, and we sought to characterize secreted signals that may contribute to disease pathology. Transcriptional profiling of senescent type II alveolar epithelial cells from mice with epithelial-specific telomere dysfunction identified the transforming growth factor-β family member, growth and differentiation factor 15 (*Gdf15*), as the most significantly upregulated secreted protein. *Gdf15* expression is induced in response to telomere dysfunction and bleomycin challenge in mice. *Gdf15* mRNA is expressed by lung epithelial cells, and protein can be detected in peripheral blood and bronchoalveolar lavage following bleomycin challenge in mice. In patients with IPF, *GDF15* mRNA expression in lung tissue is significantly increased and correlates with pulmonary function. Single-cell RNA sequencing of human lungs identifies epithelial cells as the primary source of *GDF15*, and circulating concentrations of GDF15 are markedly elevated and correlate with disease severity and survival in multiple independent cohorts. Our findings suggest that GDF15 is an epithelial-derived secreted protein that may be a useful biomarker of epithelial stress and identifies IPF patients with poor outcomes.

## INTRODUCTION

Idiopathic pulmonary fibrosis (IPF) is the most common and devastating of the idiopathic interstitial pneumonias (47). As its name suggests, the full etiology of IPF is not fully understood; however, strides have been made in recent years, largely driven by genetic association and familial studies that have focused interest on epithelial dysfunction as a primary driver of disease (12, 41). Mutations in surfactant genes, including *SFTPC*, *SFTPA1*, and *SFTPA2*, cause type II alveolar epithelial cell (AEC2)-specific injury and are responsible for 1–3% of familial pulmonary fibrosis cases (41). Mutations in genes related to telomere biology, responsible for ~30% of familial (7, 8, 13, 18, 50) and ~10% of sporadic cases (23, 46), likely limit the proliferative capacity of the epithelium and increase epithelial senescence (3, 12). The downstream signals from epithelial dysfunction that lead to organ remodeling and failure remain poorly understood.

Telomeres are DNA protein caps on the ends of chromosomes that function to maintain genome stability. Telomeres shorten each time a cell divides and limit the proliferative capacity of most somatic cells (28). Critically short telomeres trigger apoptosis or senescence, depending on the specific cellular context (20). Numerous transcriptional and phenotypic changes occur in senescent cells, including changes to the repertoire of proteins secreted, which has been termed the senescence-associated secretory phenotype (SASP) (17). While the function of all SASP-associated proteins is not known, several components are thought to promote inflammation and wound healing (19, 21).

Growth differentiation factor 15 [GDF15; also known as NAG-1 (NSAID activated gene-1) and MIC-1 (macrophage inhibitory cytokine-1)] is a divergent member of the transforming growth factor (TGF)-β family of secreted proteins (15, 54). *GDF15* has been previously reported to be a stress-induced gene that is upregulated in the context of several disease states, including heart, kidney, and liver disease (26, 33, 36, 38, 39, 42), and in response to exogenous injury (29, 58, 62). In the context of lung disease, GDF15 levels have been associated with an increased frequency of exacerbations, subclinical cardiovascular disease, declining lung function, and poor outcomes in chronic obstructive pulmonary disease (25, 31, 35, 40). GDF15 levels are elevated in systemic scleroderma patients with lung involvement and upregulated in response to bleomycin exposure in mice (37). However, *Gdf15* is dispensable for bleomycin-induced pulmonary fibrosis in mice (37). Exogenous GDF15 is sufficient to cause weight loss in mice, and GDF15-neutralizing antibodies prevent tumor-associated weight loss (32). While GDF15 has been reported to signal through the canonical TGF-β receptors, TGF-β receptors I and II (14, 32), recent reports have identified a novel high-affinity receptor glial cell-derived neurotrophic factor family receptor-α life (GFRAL) (24, 30, 44, 61). The full tissue-specific distribution of GFRAL is not known, but evidence to date suggests that its expression is limited to the brain stem (24, 30, 61).

While searching for signaling molecules that mediate inflammation in response to telomere dysfunction, we identified *Gdf15* as an epithelial-derived secreted factor. *Gdf15* is expressed in response to prosenescence and profibrotic challenges in mice. In humans, we detected markedly high levels of *GDF15* expression in blood and lung tissue from IPF patients compared with controls, with the highest levels identifying individuals with severe disease and poor outcomes. Our data suggest that *GDF15* is a novel epithelial “stress signal” and biomarker of IPF that identifies patients with severe, progressing disease.

## METHODS

### 

#### Human subjects.

All studies were approved by the relevant Institutional Review Board and the Committee for Oversight of Research and Clinical Training Involving Decedents at the University of Pittsburgh and Yale University. All subjects provided written, informed consent before enrollment in the research study. IPF subjects were recruited from the Simmons Center for Interstitial Lung Diseases at the University of Pittsburgh Medical Center. Clinical, physiologic, and high-resolution computed tomography studies of these patients supported the diagnosis of IPF. Patients fulfilled the criteria of the American Thoracic Society and European Respiratory Society for the diagnosis of IPF (9, 47). Patients with known causes of interstitial lung disease were excluded. Control patients consisted of unrelated healthy subjects, randomly recruited from the University of Pittsburgh Medical Center, and had no self-reported advanced lung diseases. Yale participants were recruited from the Yale ILD Center of Excellence and the criteria for IPF that were current at the time of enrollment (10, 47). Healthy, age-matched controls without known inflammatory or fibrotic disease were recruited from the greater New Haven community, as previously described (49). Explanted lungs were obtained from subjects undergoing lung transplantation at the University of Pittsburgh Medical Center or from The Center for Organ Recovery & Education (CORE).

#### Animal studies.

All animal studies were approved by the Institutional Animal Care and Use Committee of the University of Pittsburgh. Mice were housed at the University of Pittsburgh and given ad libitum access to food and water. Adult (8–12 wk of age) mice were treated with bleomycin (1 U/kg) via intratracheal instillation. Tracheal intubation for each mouse was confirmed by observing the oscillation of a water bubble attached to the tracheal cannula due to tidal breathing. Bleomycin was diluted in sterile saline to 50 μL and pipetted into the tracheal cannula until it was completely aspirated. Plasma, bronchoalveolar lavage (BAL), and lungs were collected on *days 3*, *7*, *14*, and *21* following bleomycin or saline administration, as described previously (5). Lung injury, fibrosis (at *days 14* and *21*), or lack thereof was confirmed by histologic analysis of the left lung from each mouse. GDF15 instillation experiments were carried out similar to bleomycin experiments. Recombinant murine GDF15 (R&D Systems) was resuspended in sterile saline and instilled directly into the trachea via intubation with an 18-gauge angiocatheter. Animals were euthanized, and BAL was collected by flushing the lung three times with 0.75 mL of saline 24, 48, and 96 h after GDF15 administration.

#### Gene expression and immunoblotting.

Gene expression data and clinical attributes were obtained as part of the Lung Genomics Research Consortium (www.lung-genomics.org). We performed a pairwise comparison of control and IPF samples for GDF15 expression from microarray data. Secretome analysis of senescent murine AEC2s was carried out on previously published microarray profiling (GEO GSE56892) (3). Briefly, lineage labeled AEC2s isolated from *Trf2^Fl/+^Rosa^mTmG/mTmG^Sftpc-CreER* and *Trf2^Fl/Fl^Rosa^mTmG/mTmG^Sftpc-CreER* mice following tamoxifen treatment by fluorescent-activated cell sorting. Purity of sorted cells was shown to be >90% by immunostaining for prosurfactant protein C in sorted cells that had been spun onto slides. Significantly upregulated genes were compared with a curated list of secreted murine proteins from the MetazSecKB database (bioinformatics.ysu.edu/secretomes). For validation expression studies on murine AEC2s, RNA was purified from cells isolated from mice with and without telomere dysfunction, as described above. For tissue studies, RNA was purified from fat, small intestine, heart, kidney, liver, lung, muscle, spleen, and thymus (*n* = 3, for all tissues). All tissues were homogenized using a Bullet Blender (Next Advance), and RNA was extracted using Trizol (Invitrogen). Medulla RNA was purchased from Zyagen. cDNA was generated using iScript cDNA synthesis kit (Bio-Rad), according to the manufacturer’s protocol. Quantitative PCR was performed on a CFX96 real-time instrument (BioRad) using Sybergreen (Bio-Rad) and primers listed in Supplemental Table S2 (All supplemental material is available at https://doi.org/10.6084/m9.figshare.8320709.). Expression changes were normalized to *Hprt* and *B2m* transcripts. Lung tissues were obtained from excess pathologic tissue after lung transplantation, as described (22). Control lungs (donor) were donated organs not suitable for transplantation from the Center for Organ Recovery & Education (CORE). Tissues were prepared for Western blotting by homogenizing in RIPA buffer containing Halt protease inhibitors (Thermo Fisher Scientific) in a bullet blender. Lysates were separated on 4–15% SDS-PAGE gels and transferred to PVDF membranes (Bio-Rad). Membranes were blocked and incubated with primary antibodies at 4°C overnight from the following sources: GDF15 (G-5, Santa Cruz Biotechnology; recognizes proGDF15), and GAPDH (FL-335, Santa Cruz Biotechnology).

#### Immunohistochemistry.

Biopsies were obtained from explanted lungs, fixed in formalin, and embedded in paraffin. Sections were deparaffinized and stained following standard procedures. Slides were stained with GDF15 antibodies (G-5, Santa Cruz Biotechnology), developed with 3, 3′-diaminobenzidine, and counterstained with hematoxylin QS, according to the manufacturer’s protocol (Vector Laboratories). Slides were scanned at HistoWiz. Differential counts were performed on BAL cells that had been spun onto slides and stained with Kwik-Diff kit, according to the manufacturer’s protocol (Thermo Fisher Scientific).

#### Luminex and ELISA assays.

Plasma samples from participating subjects were used for the Bioplex and ELISA analysis. Plasma samples were prepared from blood samples immediately after sample collection and stored at −80°C. For the first cohort, GDF15 levels were analyzed using a custom multiplex Luminex assay (R&D Systems). For the second and third cohorts, GDF15 levels were analyzed using a human GDF15 Quantikine ELISA Kit (R&D Systems), according to the manufacturer’s protocol.

#### Single-cell RNA sequencing analysis.

Single-cell RNA sequencing data were downloaded from GEO (GSE128033) (43). Three donor and three IPF samples were used in our analysis [see Morse et al. (43) for a full description of the explant samples]. Data cleaning, normalization, clustering, and cluster identification were carried out exactly as described. Violin plots were generated using the Seurat package in R.

#### Telomere length measurement.

Peripheral blood mononuclear cells were isolated from patients using Ficoll-Paque density centrifugation. Telomere length was measured using flow cytometry, combined with fluorescence in situ hybridization (flowFISH), at Johns Hopkins University, as described previously (6).

#### RNA in situ hybridization.

Probes specific for the indicated genes were purchased from Advanced Cell Diagnostics (ACD; RNAscope). Tissues were processed according to the manufacturer’s protocol. Multiplex fluorescents images were acquired on an Olympus Fluorview 1000 confocal microscope in the Center for Biologic Imaging at the University of Pittsburgh. Negative control probes were purchased from ACD. Bright-field images were acquired on a Nikon Eclipse 55i upright microscope.

#### Statistical analysis.

Natural log transformation of GDF15 was compared between groups using Welch’s *t* test. We used Pearson correlation to assess the correlation between baseline percent forced vital capacity (FVC%) and percent diffusing capacity for carbon monoxide (Dl_CO_%) scores with GDF15 concentration. Kaplan-Meier and log-rank tests were used to compare the survival function (of time to mortality or first lung transplant) between two groups of patients based on the GDF15 level and were age adjusted. We used mixed-effect models with random coefficients to assess the interaction between baseline GDF15 and follow-up duration (in years). This interaction represents the effect of baseline GDF15 in annualized rate of FVC% and Dl_CO_% change. All analyses were performed in Stata 15.0 (StataCorp, College Station, TX).

## RESULTS

### 

#### Gdf15 is upregulated in AEC2s in response to telomere dysfunction.

**Fig. 1.**
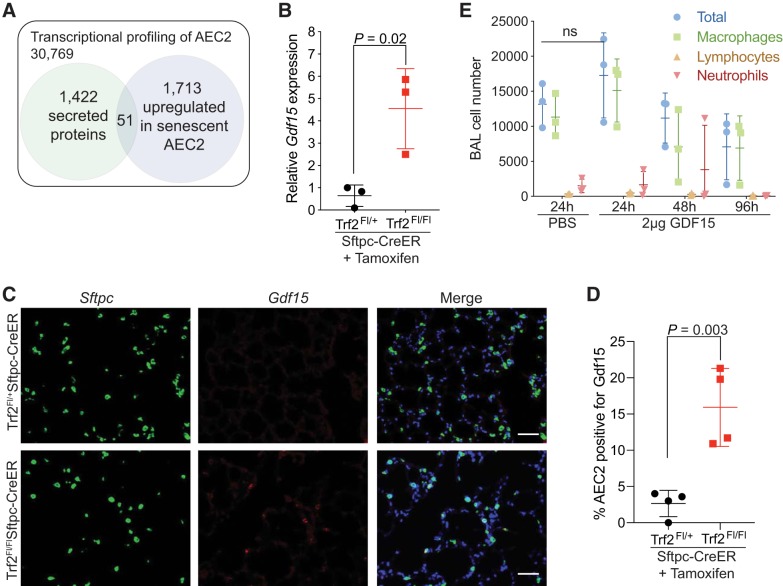
Growth and differentiation factor 15 (*Gdf15*) is upregulated in response to telomere dysfunction. *A*: schematic of our analysis strategy for identifying secreted proteins from transcriptional profiling data. Transcriptional data were obtained as described previously (3). Differentially upregulated genes that were also annotated as secreted proteins are identified. *B*: quantitative real-time PCR for *Gdf15* from sorted type II alveolar epithelial cells (AEC2s) from *Trf2^Fl/+^Sftpc-CreER* (control) and *Trf2^Fl/Fl^Sftpc-CreER* (senescent) AEC2s. Cells were sorted 10 days after treatment with tamoxifen based on green fluorescent protein expression from *mTmG* reporter allele (3). Gene expression was normalized to *Hprt* and *B2m*. *C*: representative images of RNA in situ hybridization staining for *Gdf15* from mouse lungs 6 wk after treatment with tamoxifen showing AEC2-specific expression of *Gdf15*. AEC2s were identified by expression of the *Sftpc* transcript. Scale bar is 50 µM. *D*: quantitation of the colocalization of *Gdf15* and *Sftpc* transcripts (*n* = 4 mice per group). *E*: bronchoalveolar lavage cell counts from mice treated with 2 µg of GDF15. GDF15 or sterile saline was instilled directly into the lungs and the bronchoalveolar lavage was collected thereafter at the indicated times. Total viable cells were quantitated by trypan blue staining, and a differential count was performed on >100 cells. Values are means and standard deviation (SD). Student’s *t* test (two-tailed) was used to compare groups.

We previously developed an animal model that permitted the induction of telomere dysfunction and cellular senescence specifically in AEC2s (3). Animals with AEC2-specific telomere dysfunction developed pulmonary inflammation 14–21 days following induction of telomere dysfunction. To identify AEC2-derived signals that may be responsible for recruiting inflammatory cells, we analyzed transcriptional profiling data from purified AEC2s, focusing on 1,422 curated secreted proteins from the MetazSecKB database (http://bioinformatics.ysu.edu) that could be mapped to our data. We identified 51 transcripts that encoded secreted proteins that were upregulated in response to telomere dysfunction-mediated senescence (Table 1 and Supplemental Table S1). The top transcript from this analysis mapped to *Gdf15*, and we confirmed its differential expression by quantitative PCR in purified AEC2s (Fig. 1, *A* and *B*). GDF15 was strongly expressed by airway cells by RNA in situ hybridization (RNA-ISH) and could be identified in AEC2s following induction of telomere dysfunction in *Trf2^Fl/Fl^Sftpc-CreER* mice (Fig. 1, *C* and *D*, and Supplemental Fig. S1*A*). *GDF15* has been identified in several disease contexts and has been reported to both support and inhibit inflammatory cell recruitment (14, 34, 55). We tested if GDF15 was sufficient to induce inflammation by instilling 2 µg of recombinant GDF15 via intratracheal instillation into the lungs and examined bronchoalveolar lavage (BAL) fluid 24, 48, and 96 h later. We did not observe any significant changes in the total number of specific inflammatory cell types in BAL from GDF15-instilled mice, despite the high levels of GDF15 (Fig. 1*E*). Together, these data support that *Gdf15* is upregulated in response to telomere dysfunction, but alone it is not sufficient to cause inflammation in the lung.

**Table 1.** Genes encoding secreted proteins that are transcriptionally upregulated in senescent murine type II alveolar epithelial cells

Gene	Gene Name	Fold Change	*P* Value
*Gdf15*	Growth differentiation factor 15	2.31	0.001
*Tgfbi*	Transforming growth factor, β induced	1.59	0.011
*Ssc5d*	RIKEN cDNA A430110N23 gene	1.47	0.041
*Tff2*	Trefoil factor 2 (spasmolytic protein 1)	1.46	0.041
*Cma1*	Chymase 1, mast cell	1.40	0.012
*Spon1*	Spondin 1, (f-spondin) extracellular matrix protein	1.40	0.023
*Ncan*	Neurocan	1.39	0.016
*Fetub*	Fetuin-β	1.39	0.014
*Mif*	Macrophage migration inhibitory factor	1.37	0.008
*Fibin*	Fin bud initiation factor homolog (zebrafish)	1.35	0.040
*Ang2*	Angiogenin, ribonuclease A family, member 2	1.33	0.048
*Ephx3*	Epoxide hydrolase 3	1.31	0.032
*Il17b*	Interleukin 17B	1.28	0.046
*SlpI*	Secretory leukocyte peptidase inhibitor	1.26	0.012
*Il17c*	Interleukin 17C	1.26	0.050

Fold change is relative change in expression calculated by dividing the expression in senescent type II alveolar epithelial cells (AEC2) compared with control AEC2.

#### GDF15 is expressed in response to bleomycin.

**Fig. 2.**
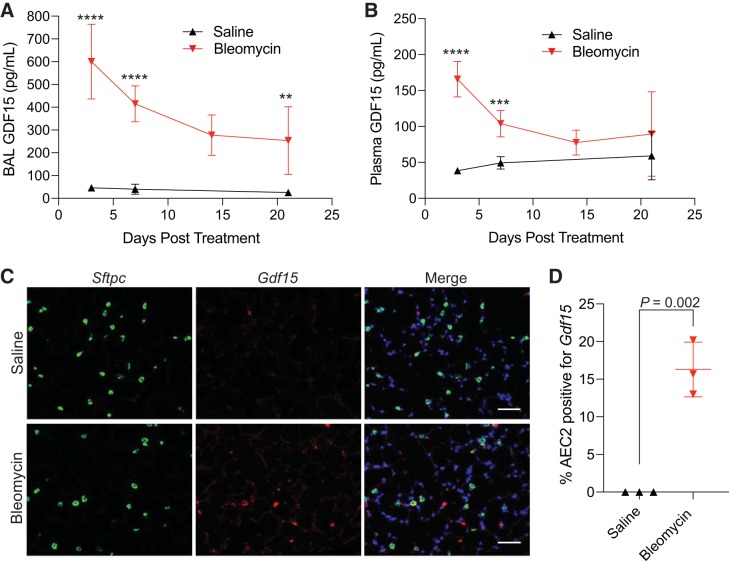
Bleomycin induces expression of growth and differentiation factor 15 (GDF15). *A* and *B*: quantitative ELISA of GDF15 levels in bronchoalveolar lavage (BAL; *A*) and plasma (*B*) from mice treated with intratracheal bleomycin or saline. Mice were treated on *day 0*, and groups of 6–7 mice (at least 3 male and 3 female at each time point) were harvested at the indicated time points. *C*: representative RNA in situ hybridization of *day 3* lungs showing alveolar expression of *Gdf15*. *D*: quantitation of colocalization of *Sftpc* and *Gdf15* transcripts in RNA in situ hybridization staining. Values are means and SD. Student’s *t* test (two-tailed) was used to compare groups. ****P < 0.0001, ***P < 0.001, and **P < 0.01.

We next explored if *Gdf15* upregulation was specific to telomere dysfunction or if additional tissue stressors would induce its expression, as has been reported (53, 58, 59, 62). We administered bleomycin, a widely used pulmonary toxin, via intratracheal instillation and examined the lungs, BAL, and plasma at several time points. BAL GDF15 protein levels were highest 3 days after bleomycin administration and remained significantly higher than in saline-treated animals (Fig. 2*A*) at all time points examined. Similarly, plasma levels of GDF15 were elevated in response to bleomycin, but returned to baseline levels after 21 days (Fig. 2*B*). *Gdf15*-expressing AEC2s could be identified in mouse lungs 3 days after treatment with bleomycin, in addition to a large number of non-AEC2s, suggesting that additional cell types express *Gdf15* (Fig. 2, *C* and *D*). These results are consistent with previous results, demonstrating that *Gdf15* expression is increased in whole lung lysates following treatment with bleomycin (37). These findings suggest that *Gdf15* expression is induced following bleomycin treatment, in addition to telomere dysfunction.

#### GDF15 is upregulated in IPF and is expressed by epithelial cells.

**Fig. 3.**
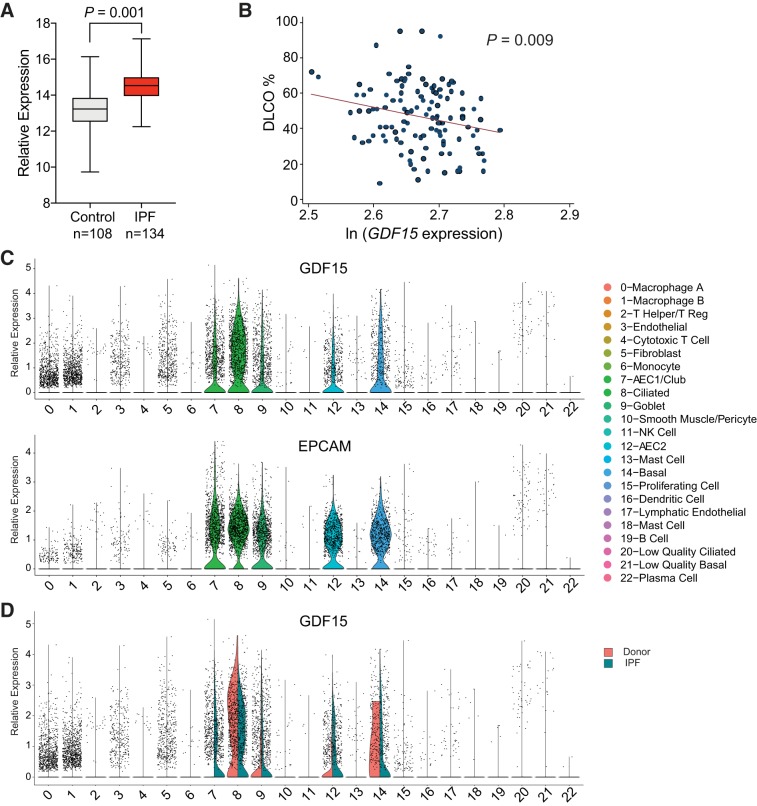
Growth and differentiation factor 15 (*GDF15*) is upregulated in idiopathic pulmonary fibrosis (IPF) and expressed by epithelial cells. *A*: box-and-whisker plot of *GDF15* expression data from the Lung Genomics Research Consortium (LGRC). Horizontal line marks the median value, box boundaries show the upper and lower quartiles, and whiskers show high and low values. Relative expression was calculated from normalized hybridization signal from microarray data. Welch’s *t* test, two tailed, was used to compare groups. *B*: correlation between natural log of *GDF15* expression from IPF patients and carbon monoxide diffusion capacity (Dl_CO_) in LGRC samples (Pearson correlation = −0.24). *C*: violin plots of *GDF15* and *EPCAM* (epithelial cell adhesion molecule) expression in scRNA-seq data demonstrating epithelial specific expression of *GDF15* (43). Data were processed and clustered exactly as described (43). The identity of each cluster is listed in the legend on the *right*. *D*: violin plot comparing *GDF15* expression in donor and IPF lungs. AEC1 and AEC2, type I and II alveolar epithelial cell, respectively; NK, natural killer.

Telomere dysfunction and bleomycin are both known to induce pulmonary fibrosis in humans (1, 11, 13). We sought to determine whether *GDF15* was upregulated in the context of IPF in clinical samples. To accomplish this, we examined gene expression data from the Lung Genomics Research Consortium (LGRC; www.lung-genomics.org), consisting of 134 IPF samples and 108 age-matched controls (51). GDF15 expression was significantly higher in IPF lung homogenates compared with controls (*P* = 0.001; Fig. 3*A*) in a pairwise comparison. Furthermore, *GDF15* gene expression was inversely related to lung function, as measured by the Dl_CO_ in IPF patients (Fig. 3*B*). Because the LGRC data are derived from whole lung homogenates, the precise cell-type responsible for *GDF15* expression is unclear from this analysis due to the heterogeneity of the biopsies. To address this question, we reanalyzed single-cell RNA sequencing (scRNA-Seq) data from three marginal donor lungs that were declined for transplantation (donor hereafter) and three explanted lungs from patients with IPF (43). *GDF15* was expressed primarily by epithelial cells, marked by *EPCAM* (epithelial cell adhesion molecule), in addition to a subset of macrophages (Fig. 3*C*). In the context of IPF, a greater proportion of AEC1, AEC2, and club cells expressed *GDF15*. However, given the decreased abundance of cells expressing markers of AEC1 and AEC2 in IPF, club, ciliated, and basal cells are likely the most significant source of *GDF15* (Fig. 3*D*) (43). Similar results were found in scRNA-seq data sets from Reyfman et al. (48) and Xu et al. (60). Together, our gene expression analysis demonstrates that *GDF15* is expressed primarily by epithelial cells, its expression is increased in IPF, and that its expression is associated with impaired gas exchange.

#### GDF15 receptor expression.

**Fig. 4.**
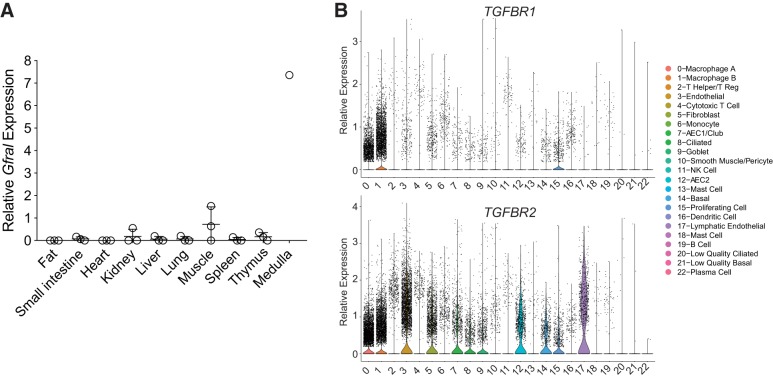
Expression of putative growth and differentiation factor 15 (GDF15) receptors. *A*: *Gfral* expression was measured in the mouse tissues shown (*n* = 3 for all tissues, except medulla for which only a single sample was measured). No signal was detected in the majority of samples, except skeletal muscle and medulla. Values are means and SD. *B*: violin plots of *TGFBR1* and *TGFBR2* (transforming growth factor-β receptors I and II, respectively) in scRNA-seq data from human lungs showing macrophage predominant expression of *TGFBR1* and broad expression of *TGFBR2* (43). AEC1 and AEC2, type I and II alveolar epithelial cell, respectively; NK, natural killer.

We next investigated the expression of GDF15 receptors to identify the potential target of this ligand. The high-affinity receptor for GDF15, GFRAL, was recently identified and shown to be expressed in the brain stem (24, 30, 44, 61). We queried our scRNA-seq data to determine whether *GFRAL* or its coreceptor *RET* was expressed in any cell types within the lung and found no evidence of expression in donor or IPF lungs (not shown). We also stained mouse lungs with antibodies to GFRAL, but found no evidence expression by immunohistochemistry (not shown). We further surveyed several mouse tissues for *Gfral* expression and, similar to previous reports (24, 30, 44, 61), only found evidence of expression in the medulla of the mouse brain (Fig. 4*A*). Previous investigations have identified the canonical TGF-β receptors, TGFBR1 and TGFBR2, as potential receptors for GDF15 (14, 32). *TGFBR1* and *TGFBR2* were broadly expressed in the human lung, with *TGFBR2* being far more abundant (Fig. 4*B*). Thus the precise cellular target of the GDF15 ligand in the lung is unclear.

#### GDF15 is expressed by epithelial cells in fibrotic regions of the lung.

**Fig. 5.**
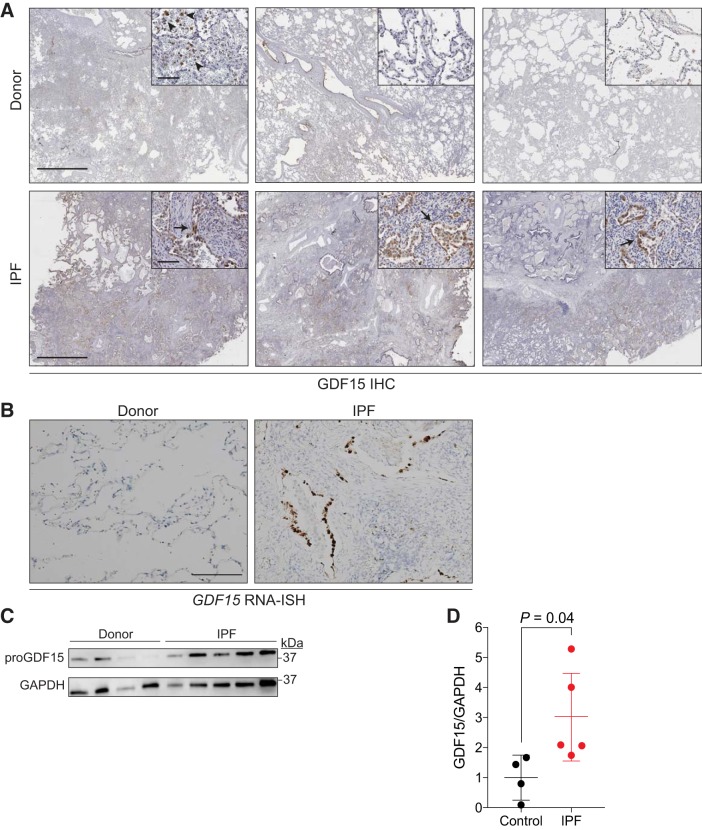
Growth and differentiation factor 15 (GDF15) is expressed by honeycomb cyst epithelial cells. *A*: representative photomicrographs from three independent donor and idiopathic pulmonary fibrosis (IPF) lungs. Slides were stained for GDF15 (brown) and counterstained with hematoxylin. GDF15 expression is present in macrophages from healthy lungs (arrowheads) but rarely in epithelial cells. In contrast, GDF15 expression was abundant in epithelial cells (arrows) and macrophages in fibrotic lungs. Scale bar in micrographs is 2 mm and 100 μm in *insets*. *B*: *GDF15* RNA in situ hybridization (RNA-ISH) in donor and IPF lungs showing epithelial-specific expression of *GDF15*. Scale bar is 100 μm. *C*: Western blot of whole lung lysate from donor and IPF lungs for proGDF15 and GAPDH as a load control. *D*: quantitation of proGDF15 in Western blot in *C*. Values are means and SD. Student’s *t* test, two tailed was used for comparison in *D*. IHC, immunohistochemistry.

We next investigated the expression of GDF15 in situ. We obtained lung tissue from donor and IPF explanted lungs and examined GDF15 expression by immunohistochemistry. GDF15 expression was localized primarily to epithelial cells in fibrotic regions of the lung and within macrophages in those areas (Fig. 5*A*). We also observed GDF15 expression in macrophages from donor lungs (Fig. 5*A*). As GDF15 is a secreted protein, we reasoned that macrophages could potentially take up GDF15 that has been secreted by other cells. To address this possibility, we performed RNA-ISH for *GDF15* in human lungs. RNA-ISH staining was limited to the epithelial cells of the lung and sparsely stained cells in the rest of the lung, suggesting that epithelial cells are the primary source of GDF15 (Fig. 5*B*). Western blotting of lung tissue samples from the lower lobe demonstrated higher expression of proGDF15 in IPF lungs (Fig. 5, *C* and *D*). These data are consistent with the mRNA expression data and demonstrate that GDF15 is expressed primarily in epithelial cells from IPF lungs.

#### Plasma GDF15 is elevated in IPF patients and correlates with disease progression.

**Fig. 6.**
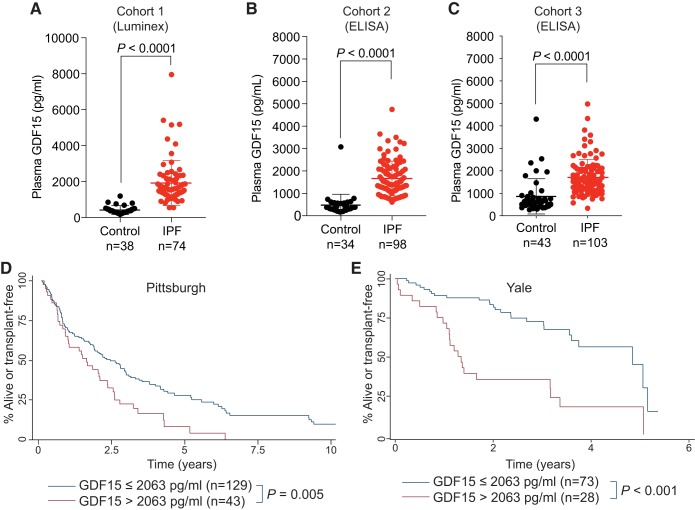
Growth and differentiation factor 15 (GDF15) is a biomarker of idiopathic pulmonary fibrosis (IPF) and identifies high-risk patients. *A–C*: plasma levels of GDF15 in three cohorts of controls and IPF patients. GDF15 was measured using Luminex assay in *cohort 1* (*A*) and ELISA in *cohorts 2* and *3* (*B* and *C*, respectively). Values are means and SD. *D* and *E*: Kaplan-Meier graph showing the proportion of patients who were alive or transplant free as a function of time. IPF patients were split into two groups based on GDF15 level. Patients from the upper quartile were compared with the lower three quartiles. Age-adjusted *P* values are from Cox regression analysis. Comparisons in *A*, *B*, and *C* used Welch’s *t* test, two tailed.

Because of our finding of increased *GDF15* expression in IPF lungs and the association with lung function, we explored if GDF15 could be detected in plasma samples from IPF patients. Using a discovery cohort of 38 control and 74 IPF patients that were approximately age and sex matched, we found significantly higher GDF15 in IPF patients compared with controls (Fig. 6*A*; 1,918 pg/mL versus 420 pg/mL, *P* < 0.0001, Welch’s *t* test). We validated these findings by examining a larger, independent cohort (34 controls and 98 IPF) utilizing an independent technique (quantitative ELISA) and found similar results with significantly higher levels of GDF15 in IPF patients (Fig. 6*B*; 1,666 pg/mL versus 475 pg/mL, *P* < 0.0001, Welch’s *t* test). We extended these findings in a third cohort from an independent medical center and found similarly elevated levels of GDF15 (Fig. 6*C*; 1,712 pg/mL versus 866 pg/mL, *P* < 0.0001, Welch’s *t* test). Pulmonary function data were available for a subset of these patients, and we explored if GDF15 correlated with lung function of IPF patients in these cohorts (Table 2). The Dl_CO_ of IPF patients was strongly and inversely correlated with GDF15 levels in all three cohorts. There was also a trend toward decreasing forced vital capacity in two of the three cohorts (Table 2). Patients with the highest plasma levels (>2,063 pg/mL; top quartile) of GDF15 had significantly shorter transplant-free survival (*P* = 0.005, University of Pittsburgh Medical Center, *P* < 0.001, Yale; Fig. 6, *D* and *E*), suggesting that high GDF15 identifies high-risk individuals. Because we identified GDF15 in the context of mice with telomere dysfunction, we examined the relationship between telomere length and GDF15 levels in a limited number of patients (*n* = 22). We did not observe any relationship between telomere length, measured by flowFISH, and plasma GDF15 levels (not shown).

**Table 2.** Clinical characteristics and correlation between plasma GDF15 and pulmonary function in patients with IPF

	Cohort 1	Cohort 2	Cohort 3
No. of samples with spirometry	62	90	103
Age (SD), yr	66.3 (8.9)	67.7 (7.7)	70.9 (6.6)
Men, *n* (%)	38 (61%)	61 (68)	79 (77)
Smoking status, *n* (%)			
No	20 (33%)	27 (30%)	27 (26%)
Former	38 (62%)	60 (68%)	73 (71%)
Current	3 (5%)	2 (2%)	3 (3%)
FVC, mean (SD), %	71 (18.7)	62 (19.5)	74 (17.0)
Dl_CO_, mean (SD), %	48 (14.3)	43 (16.1)	47 (16.2)
GAP, median (IQR)	3 (2–5)	4 (3–5)	4 (3–5)
Method for measuring GDF15	Luminex	ELISA	ELISA
Plasma GDF15 (SD), pg/ml	1,918 (1,228)	1,666 (779)	1,712 (794)
Correlation with clinical characteristics, regression β (*P* value)			
FVC%	−0.07 (0.57)	0.03 (0.78)	−0.17 (0.089)
Dl_CO_%	−0.30 (0.022)	−0.25 (0.03)	−0.23 (0.023)
GAP	0.23 (0.07)	0.09 (0.41)	0.37 (<0.001)
Change in FVC%*	−5.9 (0.083)	−3.7 (0.23)	−5.9 (0.005)
Change in Dl_CO_%*	1.2 (0.71)	3.3 (0.29)	NA

Values are means (SD). Dl_CO_, diffusing capacity for carbon monoxide; FVC, forced vital capacity; GAP, gender, age, and physiology score; GDF15, growth and differentiation factor 15; IPF, idiopathic pulmonary fibrosis; IQR, interquartile range; NA, not applicable; SD, standard deviation.

* Annualized rate of change.

## DISCUSSION

Here we identified GDF15 in an animal model of the most common genetic risk factor for IPF, telomere dysfunction, as a lung-derived secreted factor that is expressed in response to genetic and environmental stress. GDF15 is expressed primarily by epithelial cells, and its expression is elevated in IPF lungs, by whole lung gene expression analysis and protein analysis. In three independent cohorts of IPF patients, we found plasma GDF15 levels were inversely associated with diffusion capacity and declining FVC, and we identified patients with shorter transplant-free survival. Together, these findings identify GDF15 as a novel cell-specific marker of epithelial injury, as well as a novel biomarker of IPF severity.

Cellular senescence induces a constellation of changes within cells, including the activation of the senescence-associated secretory phenotype (SASP) (19). Unexpectedly, very few transcripts from previously reported SASP proteins were found upregulated in senescent AEC2 from mice. Indeed, only GDF15 and MIF (macrophage migration inhibitory factor) have been previously reported as SASP components (2, 19). This suggests that the response to senescence and the SASP may be cell-type specific. GDF15 levels have also been associated with aging (52, 57). Senescent cells accumulate with age and likely contribute to many age-associated pathologies (16). This connection would point to a novel use for GDF15 in quantifying the burden of senescent cells in the lung epithelium. In a small cohort of patients, we did not observe any relationship between telomere length and GDF15. Our data support that GDF15 is upregulated by cells undergoing a stress response, and it is possible that peripheral blood mononuclear cell telomere length may not reflect the stress status of all epithelial cells. Furthermore, telomeres are thought to function until they reach a specific threshold when their length is too short to carry out their function in suppressing the DNA damage response. Therefore, GDF15 is likely to be elevated only when telomeres reach their functional threshold length. Additional studies with larger numbers of samples are warranted to fully evaluate the relationship between telomere length and *GDF15* expression. It remains unclear what the contribution of GDF15 may be to age-associated phenotypes and how to distinguish cellular senescence from cellular stress.

We identified *Gdf15* in the context of searching for secreted factors that could be responsible for pulmonary inflammation seen in mice with telomere dysfunction (3). However, our findings suggest that GDF15 alone is not sufficient to cause inflammation in the lung. This is in contrast to several reports that have described GDF15 as a regulator of the inflammatory response (14, 59) through the canonical TGF-β-family receptors TGFBR1 and TGFBR2. While we did not observe increased inflammation in response to exogenous GDF15, we cannot draw conclusions about a potential inhibitory role for GDF15 due to the low baseline inflammation in the mouse lung. To begin to explore the downstream consequences of GDF15 signaling in the lung, we searched for cells that expressed the putative GDF15 receptors. We were unable to detect expression of the high-affinity receptor for GDF15, GFRAL, in mouse or human lungs. It is possible that GFRAL is expressed in some exceptionally rare cell types or under specific conditions (i.e., injury or additional cytokines) and that our experiments did not include these conditions. The other putative receptors for GDF15, TGFBR1 and TGFBR2, were expressed widely in the lung, including abundant expression on macrophages. However, given the recent genome-wide screens of all transmembrane proteins and failure to identify any additional receptors for GDF15 besides GFRAL (30, 44, 61), structural data supporting the unique interaction between GDF15 and GFRAL (30), and failure to identify any interaction between GDF15 and TGFβ-family receptors other than GFRAL (24, 30, 44), it is unclear what role GDF15 signaling plays locally within the lung. The downstream consequences of GDF15 signaling remain uncertain.

Numerous blood-derived biomarkers have been identified in IPF, including MMP-7, MUC-1 (KL-6), ICAM-1, IL-8, VCAM-1, SP-A, SP-D, CXCL13, CCL18, COMP, and markers of extracellular matrix turnover, among others (27, 56). Together with previous reports, our data suggest that GDF15 may be useful as a marker of epithelial injury or stress in the lung. How GDF15 could be used alone or in combination with other biomarkers to diagnose, identify distinct disease endotypes, or measure the effectiveness of clinical interventions is unknown, but merits prospective study, perhaps as a secondary end point of an IPF clinical trial.

Our study further connects telomere dysfunction with pulmonary disease, as we identified GDF15 while studying an animal model of AEC2-specific telomere dysfunction. Despite the differences between telomere dysfunction induced by deletion of TRF2 and telomere shortening in humans, modeling telomere dysfunction in mice using this model was sufficient to identify GDF15 and translate these finding into patients with IPF. While mutations in genes responsible for telomere maintenance are identifiable in a subset of cases, short telomeres are present in the majority of all IPF patients (4), supporting a strong link between telomere dysfunction and lung disease. How telomere dysfunction leads to GDF15 expression is not known; however, it likely depends on p53 signaling (45). We showed previously that telomere dysfunction can limit the capacity of the lung epithelium to proliferate and repair after injury (3, 5) and our present findings suggest that GDF15 expression represents an additional consequence of telomere dysfunction in the lung epithelium that may have systemic repercussions.

To our knowledge, this is the first study to combine an animal model, whole tissue expression data, and scRNA-Seq data to identify the source of a plasma biomarker potentially linking epithelial stress or senescence to a quantifiable and accessible surrogate. Our study is limited by its nonprospective nature; however, our findings were validated in a large, independent replication cohort and further supported by expression and in situ studies. Our findings connect one of the best know biomarkers of chronologic aging (GDF15) (52) with one of the best characterized mechanisms of aging (telomeres) in the context of an age-associated disease (IPF). We expect that these finding will spur novel investigations exploring the usefulness of GDF15 as a biomarker of sever disease and its potential role in disease pathology.

## GRANTS

This project was supported by National Heart, Lung, and Blood Institute Grants HL-113105, HL-135062 (both to J. K. Alder), HL-1226990 (D. J. Kass), HL-123766 (M. Rojas), and HL-109233 and HL-125850 (both to E. L. Herzog), funding from the Samuel and Emma Winters Foundation (J. K. Alder), funding from the Pulmonary Fibrosis Foundation (Y. Zhang), and the Dorothy P. and Richard P. Simmons Center for Interstitial Lung Disease.

## DISCLOSURES

D. J. Kass reports collaborative research funding from Regeneron Pharmaceuticals in pulmonary hypertension, which is unrelated to this article. R. Lafyatis has received consulting fees from PRISM Biolab, Merck, Bristol Myers Squibb, Biocon, Formation, Genentech/Roche, UCB, and Sanofi and grant support from Elpidera and Regeneron, not related to the submitted work. K. F. Gibson reports membership on the advisory board of Bayer Pharmaceuticals, outside the scope of the submitted work. E. L. Herzog has received grant funding from Sanofi, Bristol Myers, and Biogen, and consulting fees from Boehringer Ingelheim, Genentech, and Merck, all unrelated to the submitted work. M. Rojas reports funding from Regeneron and MedImmune, unrelated to this work. None of the other authors has any conflicts of interest, financial or otherwise, to disclose.

## AUTHOR CONTRIBUTIONS

Y.Z., J.F.M., D.J.K., and J.K.A. conceived and designed research; M.J., M.G.R., T.T., S.W., X.C., J.S., Y.C., N.C., C.R., and J.K.A. performed experiments; Y.Z., M.J., M.N., M.G.R., T.T., S.W., R.M.T., E.L.H., C.R., J.F.M., D.J.K., and J.K.A. analyzed data; Y.Z., M.N., M.G.R., S.W., E.L.H., M.R., R.L., K.F.G., J.F.M., D.J.K., and J.K.A. interpreted results of experiments; M.J., M.N., and J.K.A. prepared figures; J.K.A. drafted manuscript; Y.Z., M.N., M.G.R., R.M.T., E.L.H., M.R., R.L., D.J.K., and J.K.A. edited and revised manuscript; Y.Z., M.J., M.N., M.G.R., T.T., S.W., X.C., J.S., Y.C., N.C., R.M.T., E.L.H., C.R., M.R., R.L., K.F.G., J.F.M., D.J.K., and J.K.A. approved final version of manuscript.
